# Adult stem cell-based therapy for degenerative joint diseases

**DOI:** 10.1186/ar3594

**Published:** 2012-02-09

**Authors:** Peter G Alexander, Rocky S Tuan

**Affiliations:** 1 Center for Cellular and Molecular Engineering, University of Pittsburgh, Pittsburgh, PA 15219, USA

## 

Cell-based therapy for regenerative medicine is a major field of biomedical research including its use in the treatment of degenerative joint disease. The goal of regenerative medicine is to develop methods to repair, replace, and regenerate diseased, injured, or non-functional tissues. Towards this goal, stem or progenitor cells have been considered a highly desirable candidate cell type, because of their expandability and potential to be induced toward specific cell differentiation lineages. A key requirement in musculoskeletal tissue engineering and regeneration is that ultimately the "regenerate tissue" needs to be a three-dimensional structure. This may be accomplished through the use of engineered constructs derived by cell seeding into natural or synthetic biomaterial scaffolds. While direct cell injection is the most convenient means of cell delivery, a scaffold-based approach is capable of producing three-dimensional engineered tissues with mechanical properties compatible with those of various musculoskeletal tissues. Of the 40-50 million Americans with osteoarthritis (OA), an estimated 10-12% suffer from post-traumatic OA. We have developed an impact model for the development of post-traumatic OA. Data on the characteristics of this model in vitro and in vivo will be presented. Focal lesions developed in vivo resulting from these traumatic impacts will be repaired using stem cell-laden hydrogel or nanofiber constructs. Concurrently, cell-hydrogel and cell-nanofibrous constructs are currently being developed for the engineering of cartilaginous tissues, and information on the fabrication and biological attributes of these various tissue-engineered composites will be presented. In conclusion, tissue engineering and regenerative medicine presents an exciting, emerging inter-disciplinary research field that is a natural platform for life scientists, engineers, and clinicians working together to develop therapeutic solutions for diseased or injured tissue and organs.

## Support

Commonwealth of Pennsylvania Department of Health and the United States Department of Defense.

